# State-Specific Prevalence of Severe Obesity Among Adults in the US Using Bias Correction of Self-Reported Body Mass Index

**DOI:** 10.5888/pcd20.230005

**Published:** 2023-07-13

**Authors:** Lixia Zhao, Sohyun Park, Zachary J. Ward, Angie L. Cradock, Steven L. Gortmaker, Heidi M. Blanck

**Affiliations:** 1 Division of Nutrition, Physical Activity, and Obesity, Centers for Disease Control and Prevention, Atlanta, Georgia; 2 Center for Health Decision Science, Harvard T.H. Chan School of Public Health, Boston, Massachusetts; 3 Department of Social and Behavioral Sciences, Harvard T.H. Chan School of Public Health, Boston, Massachusetts

## Abstract

**Introduction:**

Adults with severe obesity are at increased risk for poor metabolic health and may need more intensive clinical and community supports. The prevalence of severe obesity is underestimated from self-reported weight and height data. We examined severe obesity prevalence among US adults by sociodemographic characteristics and by state after adjusting for self-report bias.

**Methods:**

Using a validated bias-correction method, we adjusted self-reported body mass index (BMI) data from the 2020 Behavioral Risk Factor Surveillance System (BRFSS) by using measured data from the National Health and Nutrition Examination Survey. We compared bias-corrected prevalence of severe obesity (BMI ≥40) with self-reported estimates by sociodemographic characteristics and state.

**Results:**

Self-reported BRFSS data significantly underestimated the prevalence of severe obesity compared with bias-corrected estimates. In 2020, 8.8% of adults had severe obesity based on the bias-corrected estimates, whereas 5.3% of adults had severe obesity based on self-reported data. Women had a significantly higher prevalence of bias-corrected severe obesity (11.1%) than men (6.5%). State-level prevalence of bias-corrected severe obesity ranged from 5.5% (Massachusetts) to 13.2% (West Virginia). Based on bias-corrected estimates, 16 states had a prevalence of severe obesity greater than 10%, a level not seen in the self-reported estimates.

**Conclusion:**

Self-reported BRFSS data underestimated the overall prevalence of severe obesity by 40% (5.3% vs 8.8%). Accurate state-level estimates of severe obesity can help public health and health care decision makers prioritize and plan to implement effective prevention and treatment strategies for people who are at high risk for poor metabolic health.

**Summary**

**What is already known on this topic?**

The prevalence of severe obesity is underestimated when self-reported weight and height data from the Behavioral Risk Factor Surveillance System (BRFSS) are used.

**What is added by this report?**

Self-reported BRFSS data significantly underestimated the prevalence of severe obesity (body mass index ≥40) among US adults compared with bias-corrected data, which provided more accurate estimates of severe obesity by sociodemographic characteristics and state.

**What are the implications for public health practice?**

State-specific accurate estimates of severe obesity provide information that public health professionals can use to prioritize effective obesity prevention and treatment strategies tailored to this population with high disease risk.

## Introduction

The high prevalence of obesity is a public health concern in the US (1,2). The prevalence of severe (or class III) obesity (body mass index [BMI, calculated as weight in kilograms divided by height in meters squared] ≥40) might be increasing faster than the overall prevalence of obesity (2,3). Many studies find that BMI has a *J*-shaped relationship to illness. In addition to the health risk of being underweight, the higher one’s BMI, the higher the risk of adverse health problems (4–6), including cardiovascular disease, type 2 diabetes, and cancers, as well as higher health care costs (7) and death (8). Findings from the 2017–2020 National Health and Nutrition Examination Survey (NHANES) using measured heights and weights indicated that almost 1 in 10 adults in the US had severe obesity (2,9). The prevalence of severe obesity almost doubled from 1999–2000 (4.7%) to 2017–2018 (9.2%) among US adults according to NHANES data (2).

NHANES data have been used for monitoring national trends in obesity prevalence (10,11). However, NHANES is not designed to estimate state-level prevalence, and the small sample size (approximately 5,000 people each year) is insufficient to generate reliable annual estimates of severe obesity prevalence for small population groups (12). The Behavioral Risk Factor Surveillance System (BRFSS) is state-based and is the largest state-representative telephone survey, collecting self-reported height and weight data from over 400,000 participants annually (13). BRFSS data have been used for monitoring annual state-specific adult obesity prevalence for the Centers for Disease Control and Prevention’s (CDC’s) obesity maps (14). The BRFSS data are essential for producing obesity prevalence estimates at the state level. However, self-reported weight and height data are subject to reporting or social desirability biases or both. These biases tend to be greater among people with higher BMI and may lead people to underreport their weight and overreport height; therefore, obesity prevalence based on self-reported data are underestimated when compared with obesity prevalence based on measured data (15).

Ward and colleagues developed a novel method to correct BMI for self-report bias and validated it against measured data from NHANES (16,17). This bias-correction method adjusted the entire BMI distribution; doing so has been shown to better capture tails of the BMI distribution and lead to more precise estimates of obesity prevalence, particularly the prevalence of a BMI of 35 or higher, compared with previous methods based on regression models (18,19) to address self-report bias. By using this bias-correction method, Ward and colleagues projected that by 2030, approximately 1 in 2 US adults will have obesity and nearly 1 in 4 adults will have a BMI of 35 or higher, and they predicted that a BMI of 35 or higher is likely to become the most common BMI category in 10 states by 2030 (16).

Although national trends of severe obesity (BMI ≥40) have been documented (2,20), little is known about state-specific prevalence of severe obesity after correcting for self-report bias. Accurate estimates of the prevalence of severe obesity at the state level help state health officials support decisions to develop and implement effective preventive policies and support treatment programs to prevent and manage severe obesity. Therefore, we examined corrected estimates of the prevalence of severe obesity by sociodemographic characteristics and by state among US adults by using the bias-correction method.

## Methods

### Data sources

We obtained data from BRFSS and NHANES. The BRFSS is a state-based random-digit–dialed telephone survey of US noninstitutionalized residents. It collects state-level data from more than 400,000 adults, aged 18 years or older, each data collection year to monitor chronic health conditions and health-related risk behaviors in all 50 states, the District of Columbia, 2 territories, and 1 freely associated state (13). Self-reported height and weight data were used to calculate BMI. Severe (also known as class III) obesity was defined as BMI of 40 or higher (21). We used BRFSS data from 2013–2018 to match with NHANES 2013–2018 data from the same period to correct for self-report bias. We excluded participants from Guam, Puerto Rico, or the Virgin Islands in BRFSS to be consistent with the sampling frame for NHANES. We sequentially excluded participants who were pregnant (n = 15,124) and those with missing sex data (n = 1,457), age data (n = 36,213), or race or ethnicity data (n = 42,201). We further excluded adults with missing data on weight and height and those with biologically implausible values (ie, height <50 cm [12 inches] or >300 cm [118 inches] and weight <20 kg [44 pounds]) (n = 164,237). This yielded 2,467,810 participants in BRFSS 2013–2018. We applied the same exclusion criteria to BRFSS 2020: we excluded 2,153 pregnant people, adults with missing age data (n = 8,203) or race or ethnicity data (n = 7,110), those with missing data on weight or height, and those with biologically implausible values (n = 33,326), which left an analytic sample of 344,087 adults. An additional 567 (0.2%) adults missing education level data, 1,277 (0.4%) adults missing marital status, and 2,012 (0.7%) adults missing employment status were excluded only from the analyses that included these variables.

NHANES uses a complex sampling procedure to collect health and nutritional data from a nationally representative sample of about 5,000 noninstitutionalized US residents of all ages each year. It combines a home interview and physical examination that collects both self-reported and measured height and weight data (12). Data on measured height and weight were pooled from NHANES 2013–2018. We excluded participants who were pregnant (n = 190) and those with missing weight data (n = 221) or height data (n = 27), and after ensuring no demographic variables of interest were missing, the final NHANES sample included 16,782 participants aged 18 years or older.

### Explanatory variables

Sociodemographic variables included sex (men, women); age group (18–34 years, 35–49 years, 50–64 years, ≥65 years); race and ethnicity (Hispanic, non-Hispanic Asian, non-Hispanic Black, Other non-Hispanic [including other race or ethnicity designation and multiracial], non-Hispanic White); education (less than high school graduate, high school graduate or General Educational Development certificate, some college, college graduate or above); marital status, dichotomized into married or domestic partnership and not married (ie, divorced, widowed, separated, or never married); employment status (employed, not employed, retired); census region (Northeast, Midwest, South, West); census division (New England, Middle Atlantic, East North Central, West North Central, South Atlantic, East South Central, West South Central, Mountain, Pacific); and locality (metropolitan counties, nonmetropolitan counties).

### Adjustment for self-report bias

We adjusted BMI in BRFSS for self-report bias by using measured data from NHANES. Adjusting self-reported BMI directly maintained the individual-level relationship between height and weight. We used a validated bias-correction method to adjust the distribution of self-reported BMI in BRFSS to be consistent with measured BMI in NHANES (16,17). Sample R (R Foundation for Statistical Computing) code to perform the bias-correction is available at https://github.com/zward/BMI-bias-correction. We estimated the quantile-specific differences between self-reported BMI in BRFSS 2013–2018 and measured BMI in NHANES 2013–2018 by sex and age group, then fit cubic splines to estimate self-report bias across the entire BMI distribution. We then calculated the (sample-weighted) quantile of the self-reported BMI for each participant in BRFSS 2020 and predicted their BMI difference by using the fitted cubic splines. Each person’s BMI was then adjusted for this self-report bias according to his or her BMI quantile. We used the 2-sample Kolmogorov–Smirnov test to ensure that the empirical cumulative distribution functions of the adjusted (BRFSS) and measured (NHANES) BMI distribution were statistically similar (*P* > .05).

### Statistical analyses

We estimated the weighted prevalence and 95% CI of self-reported or bias-corrected severe obesity (BMI ≥40) for the overall population, by sociodemographic characteristics and state. We further stratified the bias-corrected prevalence estimates of severe obesity at the state level by sex. We used *t* tests for comparing prevalence estimates within sociodemographic subgroups (eg, men vs women, 18–34 years vs 35–49 years, non-Hispanic White vs non-Hispanic Black). All tests were 2-sided, and *P* values of less than .05 were considered significant. No adjustments were made for multiple comparisons. The analyses were conducted in R and in SAS-callable SUDAAN version 11.0 (RTI International) with sample weights to account for the complex sampling design.

## Results

In 2020, the bias-corrected prevalence of severe obesity (BMI ≥40) was 8.8% among US adults, whereas the uncorrected prevalence of severe obesity based on self-reported BMI was 5.3%, which underestimated the prevalence by 40% (Table 1). Self-reported BMI data resulted in underestimation of severe obesity prevalence for all sociodemographic subgroups. The prevalence of severe obesity differed by sociodemographic subgroups regardless of bias correction. After bias correction, the prevalence of severe obesity was higher among women (11.1%), adults aged 35 to 49 (10.3%) and aged 50 to 64 (10.0%), non-Hispanic Black adults (14.6%), those who had less than a high school diploma (12.4%), those who were not married (9.8%), those who were unemployed (11.9%), those living in the Midwest (10.0%) and South (9.6%) regions and the East South Central (12.0%) division, and those living in nonmetropolitan counties (10.8%) compared with their counterparts (Table 1).

**Table 1:** Prevalence of Severe Obesity^a^ Among US Adults Aged ≥18 Years by Selected Characteristics, Behavioral Risk Factor Surveillance System, 2020

Characteristics	Respondents, n (%)^b^	Prevalence of severe obesity, % (95% CI)	
		Self-reported	Bias-corrected
**Overall**	344,087 (100)	5.3 (5.1–5.4)	8.8 (8.6–9.0)
**Sex**			
Men	164,001 (50.5)	4.2 (4.0–4.5)	6.5 (6.2–6.8)
Women	180,086 (49.5)	6.3 (6.1–6.6)	11.1 (10.8–11.5)
**Age, y**			
18–34	59,991 (29.6)	4.6 (4.3–4.9)	9.5 (9.0–10.1)
35–49	66,676 (22.9)	6.9 (6.5–7.3)	10.3 (9.8–10.9)
50–64	94,700 (25.1)	6.2 (5.7–6.6)	10.0 (9.5–10.5)
≥65	122,720 (22.4)	3.5 (3.2–3.7)	4.9 (4.6–5.2)
**Race and ethnicity**			
Hispanic	26,159 (16.3)	5.4 (4.8–6.1)	10.4 (9.5–11.4)
Non-Hispanic Asian	8,130 (5.5)	1.3 (0.9–2.1)	2.3 (1.7–3.3)
Non-Hispanic Black	26,435 (12.0)	9.6 (8.7–10.4)	14.6 (13.7–15.5)
Non-Hispanic White	266,146 (63.1)	4.7 (4.5–4.9)	7.8 (7.6–8.0)
Other Non-Hispanic^c^	17,217 (3.1)	6.1 (5.4–6.9)	9.6 (8.8–10.5)
**Education^d^**			
Less than high school graduate	21,190 (11.7)	7.7 (6.9–8.6)	12.4 (11.4–13.5)
High school graduate or equivalent	91,992 (27.8)	5.7 (5.4–6.1)	9.5 (9.1–10.0)
Some college	96,233 (31.2)	5.7 (5.4–6.1)	9.6 (9.2–10.1)
College graduate	134,105 (29.3)	3.4 (3.2–3.5)	5.8 (5.5–6.0)
**Marital status^d^**			
Married or domestic partnership	190,904 (55.2)	4.7 (4.5–4.9)	7.9 (7.6–8.2)
Not married	151,906 (44.8)	6.0 (5.7–6.3)	9.8 (9.5–10.2)
**Employment status^d^**			
Employed	173,685 (55.9)	5.1 (4.8–5.3)	8.6 (8.3–8.9)
Unemployed	64,907 (24.3)	6.9 (6.5–7.4)	11.9 (11.3–12.5)
Retired	103,483 (19.8)	3.8 (3.5–4.2)	5.6 (5.2–6.0)
**Census region**			
Northeast	64,515 (16.6)	4.3 (4.0–4.6)	7.1 (6.7–7.5)
Midwest	97,988 (21.0)	5.9 (5.6–6.1)	10.0 (9.6–10.4)
South	100,122 (38.4)	5.9 (5.6–6.2)	9.6 (9.2–10.0)
West	81,462 (24.0)	4.5 (4.0–5.0)	7.6 (7.0–8.3)
**Census division**			
New England	38,543 (4.5)	4.0 (3.6–4.3)	6.6 (6.1–7.0)
Middle Atlantic	25,972 (12.2)	4.4 (4.0–4.8)	7.3 (6.8–7.8)
East North Central	34,376 (14.4)	6.0 (5.6–6.4)	10.3 (9.7–10.8)
West North Central	63,612 (6.6)	5.6 (5.4–5.9)	9.3 (9.0–9.7)
South Atlantic	59,068 (20.4)	5.4 (5.0–5.8)	8.6 (8.1–9.1)
East South Central	18,501 (6.1)	7.3 (6.7–7.8)	12.0 (11.3–12.7)
West South Central	22,553 (11.9)	6.0 (5.4–6.6)	10.2 (9.4–11.1)
Mountain	50,992 (7.6)	4.3 (4.0–4.6)	7.3 (6.9–7.7)
Pacific	30,470 (16.4)	4.6 (3.9–5.3)	7.8 (6.9–8.8)
**Locality**			
Metropolitan	236,305 (85.0)	5.0 (4.8–5.2)	8.4 (8.2–8.7)
Nonmetropolitan	107,782 (15.0)	6.8 (6.5–7.2)	10.8 (10.4–11.2)

a Defined as body mass index (calculated as weight in kilograms divided by height in meters squared) ≥40.

b n is the unweighted sample size; % is the weighted percentage.

c Includes other race or ethnicity designation and multiracial.

d Missing data: education (n = 567; 0.2%), marital status (n = 1,277; 0.4%), employment status (n = 2,012; 0.7%).

By states, self-reported prevalence of severe obesity (BMI ≥40) ranged from 3.3% (Massachusetts and Colorado) to 8.3% (West Virginia), while bias-corrected prevalence of severe obesity ranged from 5.5% (Massachusetts) to 13.2% (West Virginia) (Table 2). Seventeen states had a prevalence of severe obesity significantly higher than the national average; 16 states had a prevalence of severe obesity greater than 10%, a level not seen in the self-reported estimates. After stratifying by sex, women had higher corrected prevalence of severe obesity than men in each state. The 5 states with the highest prevalence of severe obesity among women were Mississippi (17.4%), Alabama (16.9%), West Virginia (16.2%), Arkansas (16.1%), and Louisiana (16.1%), and among men, West Virginia (10.3%), Indiana (8.7%), Georgia (8.5%), Louisiana (8.5%), and Kentucky (8.4%) (Table 2). The 2020 severe obesity maps show that self-reported BMI underestimated the prevalence of severe obesity among US adults in each state compared with bias-corrected prevalence of severe obesity (Figure).

**Table 2:** Prevalence of Self-Reported and Bias-Corrected Severe Obesity^a^ Among US Adults Aged ≥18 Years, by State and Sex, Behavioral Risk Factor Surveillance System, 2020

Location	Self-reported prevalence, % (95% CI)	Bias-corrected prevalence, % (95% CI)		
		Total	Men	Women
Alabama	7.4 (6.5–8.5)	12.7 (11.5–14.0)^b^	8.2 (6.8–9.9)	16.9 (15.1–18.9)
Alaska	4.9 (3.8–6.3)	9.2 (7.7–11.1)	7.8 (5.8–10.4)	10.9 (8.7–13.7)
Arizona	4.6 (4.0–5.3)	7.8 (7.0–8.6)	5.8 (4.9–6.9)	9.8 (8.5–11.2)
Arkansas	6.5 (5.5–7.6)	11.8 (10.5–13.2)^b^	7.6 (6.1–9.4)	16.1 (14.0–18.4)
California	4.6 (3.8–5.7)	8.0 (6.7–9.4)	6.4 (4.9–8.4)	9.5 (7.8–11.6)
Colorado	3.3 (2.9–3.8)	5.7 (5.2–6.3)	3.8 (3.2–4.5)	7.7 (6.8–8.8)
Connecticut	4.5 (3.8–5.3)	7.3 (6.4–8.3)	5.5 (4.5–6.9)	9.0 (7.6–10.7)
Delaware	6.5 (5.4–7.8)	10.2 (8.9–11.8)^b^	8.2 (6.4–10.5)	12.1 (10.2–14.3)
District of Columbia	4.0 (3.1–5.2)	7.1 (5.9–8.6)	3.9 (2.6–5.8)	10.1 (8.1–12.5)
Florida	4.5 (3.7–5.5)	6.9 (5.9–8.0)	5.1 (4.1–6.4)	8.7 (7.1–10.6)
Georgia	6.4 (5.5–7.4)	10.2 (9.1–11.4)^b^	8.5 (6.9–10.4)	11.9 (10.4–13.5)
Hawaii	3.5 (2.9–4.1)	6.1 (5.4–6.9)	5.3 (4.3–6.4)	6.9 (5.9–8.1)
Idaho	4.4 (3.7–5.2)	8.1 (7.1–9.2)	6.4 (5.2–7.8)	9.9 (8.3–11.7)
Illinois	4.7 (3.7–5.8)	8.8 (7.4–10.4)	6.5 (5.1–8.4)	11.0 (8.8–13.6)
Indiana	6.9 (6.2–7.7)	11.2 (10.4–12.2)^b^	8.7 (7.6–9.9)	13.9 (12.5–15.4)
Iowa	6.4 (5.8–7.0)	10.6 (9.8–11.4)^b^	7.7 (6.8–8.7)	13.7 (12.4–15.0)
Kansas	5.6 (5.1–6.2)	9.7 (9.0–10.5)^b^	6.8 (5.9–7.7)	12.8 (11.6–14.1)
Kentucky	7.8 (6.7–9.0)	11.8 (10.4–13.4)^b^	8.4 (7.0–10.2)	15.2 (13.1–17.6)
Louisiana	7.2 (6.2–8.3)	12.4 (11.0–13.8)^b^	8.5 (6.8–10.5)	16.1 (14.2–18.3)
Maine	5.1 (4.4–5.9)	8.3 (7.5–9.3)	6.8 (5.8–8.1)	9.9 (8.6–11.3)
Maryland	4.9 (4.4–5.5)	8.0 (7.4–8.8)	5.5 (4.7–6.5)	10.6 (9.5–11.7)
Massachusetts	3.3 (2.7–4.0)	5.5 (4.8–6.3)	4.3 (3.4–5.3)	6.8 (5.6–8.2)
Michigan	6.5 (5.8–7.3)	10.8 (9.9–11.9)^b^	7.6 (6.4–8.9)	14.2 (12.6–15.9)
Minnesota	4.5 (4.1–5.0)	7.8 (7.3–8.4)	6.4 (5.7–7.2)	9.4 (8.6–10.3)
Mississippi	7.8 (7.0–8.7)	13.0 (11.9–14.1)^b^	8.3 (7.0–9.8)	17.4 (15.8–19.1)
Missouri	6.6 (5.9–7.3)	10.4 (9.6–11.3)^b^	7.2 (6.2–8.4)	13.7 (12.4–15.2)
Montana	3.9 (3.2–4.6)	6.9 (6.1–7.8)	4.7 (3.8–5.9)	9.2 (7.9–10.7)
Nebraska	5.2 (4.7–5.8)	8.8 (8.1–9.5)	5.9 (5.1–6.7)	11.8 (10.6–13.1)
Nevada	5.0 (4.0–6.3)	8.1 (6.8–9.7)	6.5 (4.9–8.6)	9.7 (7.7–12.2)
New Hampshire	5.0 (4.2–5.9)	7.9 (7.0–9.0)	6.0 (4.8–7.5)	10.0 (8.5–11.6)
New Jersey	3.8 (3.4–4.4)	6.5 (5.8–7.2)	5.4 (4.6–6.3)	7.6 (6.6–8.7)
New Mexico	4.5 (3.7–5.4)	7.9 (6.8–9.1)	6.0 (4.7–7.5)	9.9 (8.2–11.9)
New York	4.0 (3.5–4.6)	6.8 (6.1–7.5)	5.1 (4.3–6.2)	8.5 (7.5–9.5)
North Carolina	5.2 (4.4–6.1)	9.0 (8.0–10.1)	6.8 (5.5–8.3)	11.2 (9.8–12.9)
North Dakota	5.1 (4.2–6.2)	8.4 (7.2–9.8)	6.3 (5.0–8.0)	10.8 (8.8–13.2)
Ohio	6.7 (6.1–7.3)	11.3 (10.5–12.1)^b^	7.6 (6.7–8.6)	14.9 (13.7–16.2)
Oklahoma	7.7 (6.8–8.7)	11.8 (10.6–13.1)^b^	8.2 (6.9–9.8)	15.4 (13.6–17.4)
Oregon	4.4 (3.7–5.1)	7.4 (6.6–8.3)	4.9 (4.0–6.1)	10.0 (8.7–11.5)
Pennsylvania	5.3 (4.5–6.1)	8.5 (7.5–9.6)	6.5 (5.3–8.0)	10.6 (9.1–12.2)
Rhode Island	3.8 (3.1–4.7)	7.1 (6.1–8.3)	4.9 (3.6–6.5)	9.4 (7.8–11.2)
South Carolina	6.8 (5.8–7.9)	10.1 (8.8–11.4)^b^	6.2 (4.9–7.8)	13.8 (11.9–16.0)
South Dakota	4.8 (4.0–5.9)	7.8 (6.6–9.2)	6.4 (5.0–8.1)	9.4 (7.3–12.0)
Tennessee	6.6 (5.6–7.7)	11.1 (9.8–12.5)^b^	7.8 (6.3–9.6)	14.3 (12.4–16.5)
Texas	5.4 (4.7–6.4)	9.4 (8.3–10.6)	7.1 (5.7–8.8)	11.8 (10.2–13.6)
Utah	4.7 (4.1–5.2)	7.7 (7.1–8.5)	5.8 (5.0–6.7)	9.9 (8.8–11.1)
Vermont	3.8 (3.2–4.6)	6.4 (5.6–7.3)	5.0 (4.0–6.2)	7.9 (6.7–9.3)
Virginia	5.4 (4.8–6.2)	8.8 (8.0–9.7)	6.4 (5.4–7.5)	11.3 (10.0–12.7)
Washington	4.4 (3.9–4.9)	7.2 (6.6–7.9)	5.4 (4.7–6.3)	9.1 (8.2–10.2)
West Virginia	8.3 (7.4–9.3)	13.2 (12.1–14.4)^b^	10.3 (8.9–12.0)	16.2 (14.5–18.0)
Wisconsin	5.4 (4.5–6.4)	9.3 (8.1–10.6)	6.0 (4.8–7.5)	12.8 (10.8–15.0)
Wyoming	3.6 (2.8–4.4)	6.9 (5.8–8.2)	4.8 (3.6–6.2)	9.4 (7.6–11.6)

a Defined as body mass index (calculated as weight in kilograms divided by height in meters squared) ≥40.

b States had significantly higher bias-corrected estimates than the national average estimate (8.8% [95% CI, 8.6%–9.0%]).

**Figure:**
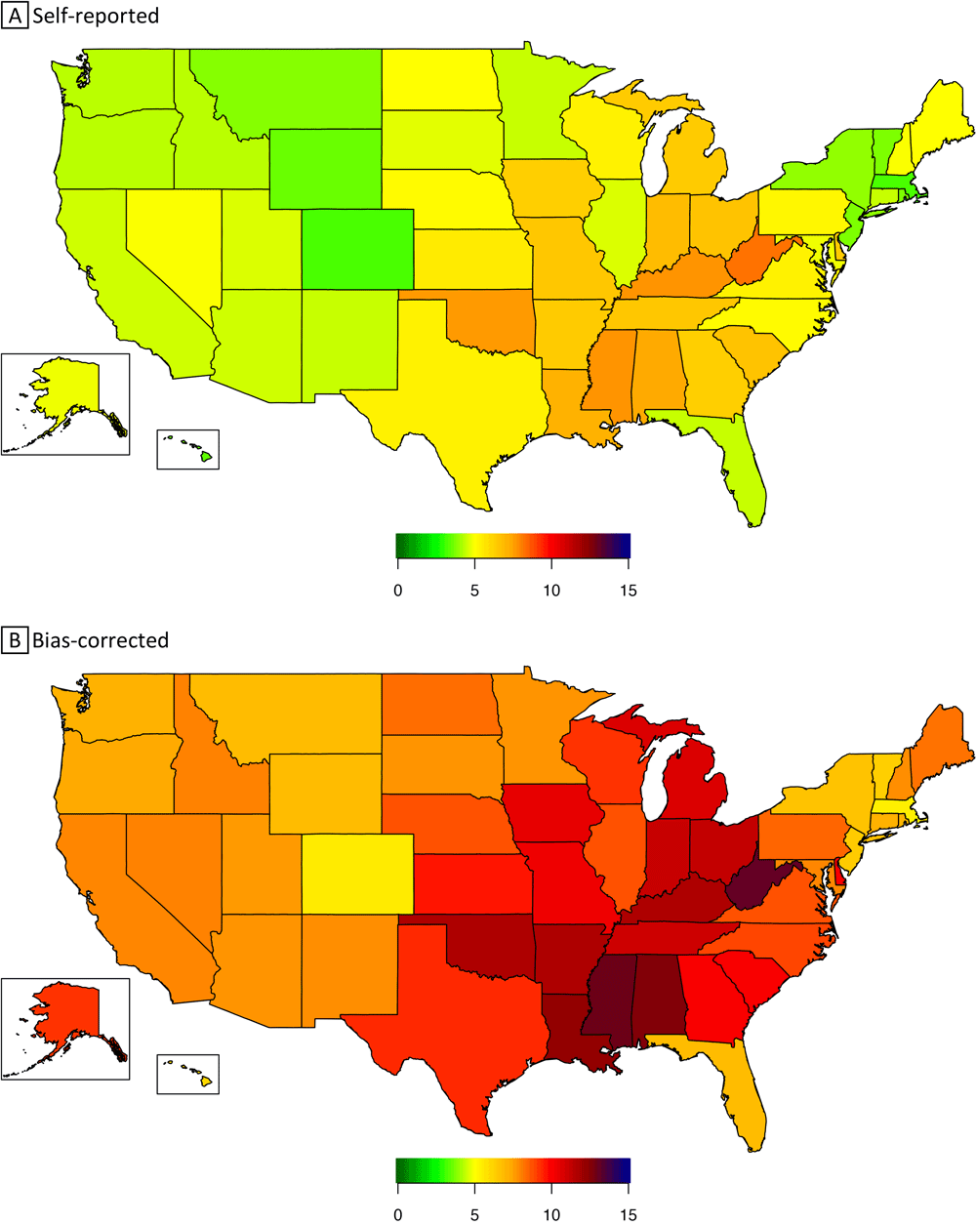
Prevalence of self-reported (panel A) and bias-corrected (panel B) severe obesity (body mass index ≥40) among US adults, by state, Behavioral Risk Factor Surveillance System, 2020. Body mass index is calculated as weight in kilograms divided by height in meters squared.

## Discussion

Using a novel bias-correction method, we found that 8.8% of US adults had severe obesity (BMI ≥40) based on bias-corrected BMI, whereas 5.3% had severe obesity based on self-reported BMI in 2020, which underestimated the prevalence by 40%. There were disparities in severe obesity prevalence by sociodemographic groups. For example, the prevalence of bias-corrected severe obesity was higher among women, middle-aged adults, non-Hispanic Black adults, people with less than a high school diploma, unmarried adults, unemployed adults, adults living in the Midwest and South census regions and the East South Central division, and those living in nonmetropolitan counties.

Consistent with our findings, a previous NHANES study (based on measured height and weight) reported that 9.0% of US adults had severe obesity, and women had a higher prevalence of severe obesity (11.4%) than men (6.4%) in 2017–March 2020 (9). Other studies also found that the prevalence of severe obesity varied by sex, age group, race, Hispanic origin, and education level, similar to findings in our study (10,11,22,23). Additionally, a study using NHANES 2013–2016 data reported higher prevalence of severe obesity (BMI ≥40) among US adults living in nonmetropolitan areas compared with those living in large metropolitan areas (22). To our knowledge, our study is the first to report the prevalence of severe obesity (BMI ≥40) by marital status, employment status, and census regions and divisions of residence among US adults. Previous BRFSS studies reported that adult obesity was most prevalent in the Midwest and South regions (14,24,25) and the East South Central division (25), which follows the pattern of the regional differences we observed in severe obesity prevalence. Furthermore, we found that state-level bias-corrected prevalence of severe obesity ranged from 5.5% to 13.2%. We also found that over 1 in 10 adults were estimated to have severe obesity in 16 states, and the estimates in 17 states were greater than the national average. These geographic differences in severe obesity could be partially due to demographic changes, state-level policies, and environmental factors such as differences in social drivers including education and economic opportunities, health care policies, culture, access to healthy foods, and safe neighborhoods for physical activity (26–28).

We used the bias-correction method developed by Ward and colleagues (16). Ward and Gortmaker evaluated the method by performing cross validation using data from NHANES 1999 to 2018 and showed that it produces unbiased estimates of mean BMI and obesity prevalence, both overall and by sociodemographic subgroups (17). In addition, the method was developed to adjust the entire BMI distribution to be similar with measured BMI, thus better capturing the tails of the BMI distribution (16,17). By analyzing 2013 BRFSS data with a similar method, Ward and colleagues reported that adjustment of the entire BMI distribution produced more accurate national and state-specific estimates of obesity prevalence, especially for BMI ≥35 (29). These approaches are similar to a percentile-based method independently developed by Courtmanche et al, highlighting the intuitive appeal of quantile-specific approaches to correct for self-report bias (30). In contrast, previous regression-based approaches significantly underestimated the national prevalence of both obesity (BMI ≥30) and severe obesity (BMI ≥35) compared with measured estimates from NHANES (29). In our study, we expanded previous efforts to estimate the prevalence of severe obesity (class III obesity, BMI ≥40). We confirmed that the method produced national estimates of severe obesity that were comparable with national estimates from NHANES.

A major strength of our study is using BRFSS data that are representative of each state and include a large sample size. BRFSS has been used to monitor state-specific adult obesity prevalence to provide information for state and local public health agencies (14). Nevertheless, this study has certain limitations. Although we adjusted self-reported BMI distribution against measured BMI from NHANES, the measured data were pooled from multiple years of NHANES data to obtain sufficient sample size for the bias correction. We therefore cannot capture shifts in BMI distribution that may have occurred during this period. However, previous analysis of NHANES found no significant changes of severe obesity prevalence during this time (2), suggesting that pooling across recent years would not substantially affect our results.

Severe obesity (BMI ≥40) remains a serious public health concern, and about 1 in 11 US adults had severe obesity in 2020. Severe obesity prevalence varied widely by sociodemographic groups and state. Self-reported BRFSS data underestimated the prevalence of severe obesity. In the absence of state representative surveys with measured BMI at the state level, the bias-correction method we used provides the best available option to produce corrected estimates of state-specific severe obesity among US adults. Given that adults with severe obesity have greater risk of comorbidities, premature death, and health care costs than the people with moderate obesity, accurately monitoring the prevalence of severe obesity at the state level can support state public health, health care systems, and other decision makers in prioritizing preventive policies and treatment programs, including weight-management programs, weight-loss medications, and bariatric surgery, to support people with severe obesity and to help mitigate further disease risk.
